# *CD28* Genetic Variants Increase Susceptibility to Diabetic Kidney Disease in Chinese Patients with Type 2 Diabetes: A Cross-Sectional Case Control Study

**DOI:** 10.1155/2021/5521050

**Published:** 2021-04-17

**Authors:** Yangyang Li, Li Jin, Jing Yan, Hong Zhang, Rong Zhang, Cheng Hu

**Affiliations:** ^1^Shanghai Diabetes Institute, Shanghai Key Laboratory of Diabetes Mellitus, Shanghai Clinical Centre for Diabetes, Shanghai Jiao Tong University Affiliated Sixth People's Hospital, Shanghai, China; ^2^Institute for Metabolic Disease, Fengxian Central Hospital Affiliated to Southern Medical University, Shanghai, China

## Abstract

Few studies have illuminated the genetic role of T cell costimulatory molecule *CD28/CD80/CTLA4* variants in diabetic kidney disease (DKD) susceptibility. We aimed to investigate the causal role of genetic polymorphisms in *CD28/CD80/CTLA4* with DKD susceptibility in patients with T2DM. A total of 3253 patients with T2DM were recruited for genotyping: including 204 DKD patients and 371 controls in stage 1 and 819 DKD patients and 563 controls in stage 2; besides, 1296 T2DM patients were selected for the analysis of association between loci and DKD-related traits. A subset of 227 T2DM patients (118 patients with DKD and 109 patients without DKD) from the total population above were selected to assess serum soluble CD28 (sCD28) levels. Then, we performed a candidate gene association study to identify single-nucleotide polymorphisms (SNPs) associated with DKD susceptibility and further used those SNPs to perform Mendelian randomization analyses of serum sCD28 level and DKD susceptibility. Under additive genetic models, *CD28*-rs3116494 (OR = 1.29 [95% CI 1.11, 1.51], *P* = 0.0011) and *CD80*-rs3850890 (OR = 1.16 [95% CI 1.02, 1.31], *P* = 0.0283) were associated with DKD susceptibility adjusted for age, gender, body mass index (BMI), duration of diabetes, and HbA1c. *CD28*-rs3116494 was associated with serum sCD28 level (*β* = 0.26 [95% CI 0.08, 0.44], *P* = 0.0043). The Mendelian randomization analysis showed that *CD28*-rs3116494 played a causal role in DKD by influencing serum sCD28 levels (*β* = 1.15 [95% CI 0.46, 1.83], *P* = 0.0010). In conclusion, we identified that two novel SNPs, *CD28*-rs3116494 and *CD80*-rs3850890, were associated with DKD susceptibility. Using the Mendelian randomization analysis, our study provided evidence for a causal relationship between serum CD28 levels and DKD with T2DM in the Chinese population.

## 1. Introduction

Diabetic kidney disease (DKD) is a major microvascular complication of diabetes mellitus (DM), which remains a leading cause of end-stage renal disease (ESRD) and an independent risk factor for all-cause mortality [1–3]. The prevalence of DKD in type II DM (T2DM) is approximately 30-50% among US diabetic adults [4]. It is recognized that intensive glucose control reduces the risk of DKD [5]. Despite advances in glycemic control therapies, the development of ESRD remains a growing problem globally, and it has generated a substantial public health burden over the past 2 decades [2, 6]. However, under similar durations of diabetes and comparable blood glucose concentrations, the occurrence, progression, and regression of DKD are highly variable in T2DM [7]. The Diabetes Control and Complications Trial showed that intensive glycemic control does not prevent from DKD in some patients, while others with poor glycemic control did not develop DKD [8, 9]. Other risk factors for DKD in diabetics should not be ignored, including gender, obesity, hypertension, inflammation, resistance to insulin, hypovitaminosis D, and dyslipidaemia [10]. Moreover, a hereditary component to DKD has been recognized for a long time, and many genetic loci in specific genes have been identified to be associated with DKD. Patients with different genetic backgrounds have different metabolic, immunologic, and hemodynamic features, which ultimately leads to discrepancies in disease susceptibility [8, 11, 12].

Recent studies have reported increased macrophage and T lymphocyte infiltration in patients with DKD [13–16]. This emphasized the possibility that activated T lymphocytes, macrophages, and related immunologic responses were associated with DKD. Elevated levels of plasma inflammatory cytokines, such as tumor necrosis factor-*α*, interleukin-1*β*, and monocyte chemoattractant protein-1, were also observed in animal models and patients with DKD [17, 18]. Immunological responses are initiated by T lymphocyte activation through major histocompatibility complexes (MHCs) and costimulatory molecules. The costimulatory molecules are important signals to elicit an effective immune response. CD28, which interacts with B7-1 (CD80) and B7-2 (CD86), is crucial for T cell proliferation, differentiation, and survival. Cytotoxic T lymphocyte-associated antigen-4 (CTLA4) that is expressed on T helper cells negatively regulates T cell activation [19–21]. Recent studies have demonstrated that aberrant production of CD28 was associated with autoimmune diseases, suggesting that the presence of aberrant costimulatory molecules might lead to immunopathogenesis in allergic inflammation and related diseases [20, 22, 23].

Additionally, B7-1, as a novel biomarker for podocyte damage, was suggested to be a potential therapeutic target for most chronic kidney diseases, and a clinical trial for B7-1 blockade in nephrotic syndrome patients is currently ongoing [24–26]. Fiorina et al. showed that high glucose levels in DKD upregulated B7-1 expression in podocytes *in vitro and in vivo*. Further, podocyte B7-1 expression was only observed in an early phase of DKD in patients with T2DM and absent in those without DKD and individuals with diffused glomerulosclerosis. They suggested B7-1 to be a feature of a subgroup of patients with T2DM exhibiting progressive decline in glomerular filtration rate and proteinuria. Circulating T cells can be active and can exacerbate podocyte injury via the CD28/B7-1 pathway. Moreover, they proposed that Abatacept, a T cell costimulation inhibitor, could improve DKD in animal models [23, 27]. However, contradictory reports showed that B7-1 cannot be induced in podocytes from patients with DKD and, in animal models, was not a therapeutic target for DKD [26, 28]. In light of these controversial reports on the role of the CD28/B7-1 pathway in DKD, population studies based on genetics could be useful in identifying the role of these costimulatory molecules in humans. Therefore, we aimed to investigate the causal relationship between circulating soluble CD28 (sCD28) and DKD in individuals with CD28-related genetic polymorphisms.

## 2. Materials and Methods

### 2.1. Participants and Phenotype Definition

We recruited 3253 patients with T2DM from the Department of Endocrinology and Metabolism in Shanghai Jiao Tong University Affiliated Sixth People's Hospital, Shanghai, China. T2DM was diagnosed using the oral glucose tolerance test based on the World Health Organization criteria: fasting plasma glucose ≥ 7 mmol/L or a 2 h plasma glucose after an oral glucose tolerance test ≥ 11.1 mmol/L. Patients with type 1 diabetes, maturity-onset diabetes of the young, mitochondrial diabetes, infections, abnormal liver function, cancer, and a medical history of other renal diseases were excluded. DKD was diagnosed as urinary albumin excretion rate (uAER) ≥ 30 mg/24 h or estimated glomerular filtration rate (eGFR) ≤ 60 mL/(min · 1.73 m^2^) in patients with T2DM.

Patients with T2DM (duration ≥ 10 years) without diabetic retinopathy (DR) and impaired kidney function (uAER < 30 mg/24 h or estimated glomerular filtration rate (eGFR) > 90 mL/(min · 1.73 m^2^)) were considered controls. Thus, the control subjects in the present study had a higher duration of diabetes than those with DKD. Additionally, control individuals had eGFR ≥ 90 mL/(min · 1.73 m^2^) and uAER ≤ 30 mg/24 h. This study was conducted in two stages: in stage 1, 575 patients with T2DM, including 204 with DKD and 371 controls, were recruited; in stage 2, 1382 patients with T2DM, including 891 with DKD and 563 controls, were recruited. Furthermore, we recruited an additional 1296 unselected patients (in these patients, the criteria for inclusion/exclusion, such as duration of diabetes, DR, eGFR, and uAER, were not considered or met) with T2DM in stage 2 for analysis of renal function-related traits to avoid discontinuous traits.

### 2.2. Clinical Measurement

The anthropometric data of all 3253 patients were recorded, including age, sex, height, weight, and T2DM duration. The body mass index (BMI) was calculated as weight (kg)/height^2^. Fasting venous blood samples were collected for DNA extraction, and serum samples were isolated and stored at -80°C until use. Clinical parameters, such as serum creatinine and HbA1c, were measured. DR was diagnosed by fundus photography performed using a 45-degree 6.3-megapixel digital nonmydriatic camera (Canon CR6-45NM, Lake Success, NY, USA). The CKD Epidemiology Collaboration equation was used to calculate eGFR [29].

### 2.3. Single-Nucleotide Polymorphism (SNP) Selection and Genotyping

In stage 1, 26 SNPs in *CD28*, *CTLA4*, and *CD80* gene regions were genotyped in patients with and without DKD as a case-control study (*n* = 575 [204 patients with DKD and 371 patients without DKD]), according to the Hapmap3 project and 1000 genome project [30, 31]. The SNPs (rs3116494 and rs3850890) that showed marginal association with DKD (*P* < 0.1) were further genotyped in stage 2 (case-control group, *n* = 1382 [819 patients with DKD and 563 patients without DKD]). Another 1296 patients with T2DM were recruited for DKD-related trait analysis.

SNPs were genotyped using a MassARRAY system (MassARRAY Compact Analyzer, Sequenom, San Diego, CA, USA). Quality controls were conducted in genotyping data, including SNP concordance rate in duplicated sample ≥ 99%, sample call rate > 90%, SNP call rate > 95%, and the Hardy–Weinberg equilibrium test (Hardy–Weinberg proportions were rejected at *P* < 0.05).

### 2.4. Serum sCD28 Measurement

A subset of 227 T2DM patients (118 patients with DKD and 109 patients without DKD) from the total population above were selected to determine serum sCD28 levels by the enzyme-linked immunosorbent assay (ELISA) using the Human CD28 ELISA kit (catalogue RAB1327; Sigma-Aldrich, Inc., St. Louis, MO, USA) according to the manufacturer's instructions.

### 2.5. Statistical Analyses

Statistical analyses were performed using SAS 9.3 (SAS Institute, Cary, NC, USA). Genetic epidemiologic analyses were performed using PLINK [32] (version 1.9) and R (version 3.6.3). Normality tests were performed, and skewed quantitative traits were log transformed. Logistic regression was used to compare the genotype distributions between DKD and control groups under an additive model adjusted for confounding factors. Pearson's correlation analysis and Spearman's rank correlation test were used to analyze the association between serum sCD28 levels and different traits. Student's *t*-tests and one-way analysis of variance tests were used to compare serum sCD28 levels between individuals with different genotypes. Multiple linear regression was used to analyze the continuous traits. Odds ratios (OR) or *β*s with 95% confidence interval (CI) were presented in reference to the minor alleles. Combined analysis of the two study stages was conducted by comprehensive meta-analysis (v2.2.057). Fixed or random effects models were chosen after testing for heterogeneity by Cochran's *Q* test. The R package MendelianRandomization was used to perform Mendelian randomization analyses using the inverse-variance weighted method [33]. According to the statistical power determined by the Quanto 1.2 software, our total sample had >90% power to detect an estimated effect size (OR = 1.3) of variants with a minor allele frequency of 0.1 at a 0.05 significance level for rs3116494 and estimated effect size (OR = 1.1) of variants with a minor allele frequency of 0.45 at a significance of 0.05 for rs3850890. The sample size used for the detection of serum sCD28 (*n* = 227) had 95% power to detect the medium effect size (0.5) based on two-sided *t*-tests with 0.05 significance level, as determined by the G power 3.1 software.

## 3. Results

### 3.1. Clinical Characteristics

The demographic and clinical characteristics of all patients are presented in Table 1. In stage 1, patients in the control group had longer T2DM durations (*P* < 0.0001) and higher HbA1c levels (*P* = 0.0505). Similar to stage 1, in stage 2 as well, the control group had longer T2DM durations (*P* < 0.0001) and higher HbA1c levels (*P* < 0.0001).

### 3.2. Association of CD28-Related Genetic Variants with DKD

**Figure 1:**
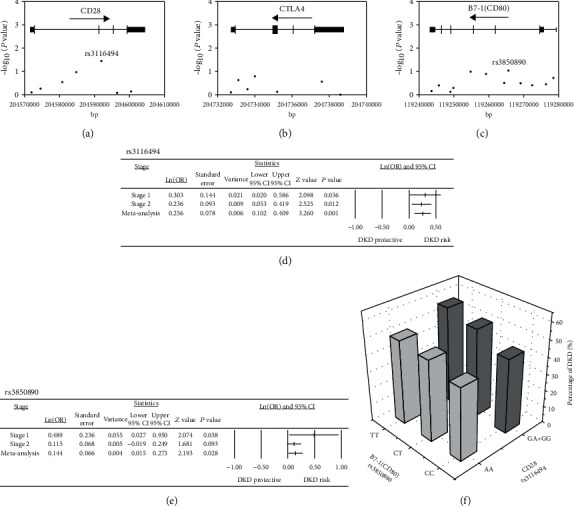
Genetic association analysis between *CD28*, *CTLA4*, and *CD80* variants with DKD in the study population. Summary of association analyses for single-nucleotide polymorphisms (SNPs) in (a) *CD28* (7 SNPs), (b) *CTLA4* (7 SNPs), and (c) *CD80* (12 SNPs) gene regions with DKD. Meta-analysis of the two study stages for association of DKD with (d) *CD28*-rs3116494 and (e) *CD80*-rs3850890. Data were natural log-transformed and show odds ratio (point), standard error, variance, lower 95% confidence interval, and upper 95% confidence interval. (f) Interaction analysis for *CD28*-rs3116494 and *CD80*-rs3850890 in the study population; the 2 SNPs were interactive (*P* < 0.01). The percentage of patients with DKD based on the genotypes for *CD28*-rs3116494 and *CD80*-rs3850890 is shown.

In stage 1, we genotyped the 7, 7, and 12 SNPs in *CD28*, *CTLA4*, and *CD80* gene regions, respectively. The base positions of SNPs are shown in Figures 1(a)–1(c). Associations between the 26 SNPs studied in stage 1 and DKD, after adjusting for age, gender, BMI, duration of diabetes, and HbA1c levels, are shown in Supplementary Table 1 and Figures 1(a)–1(c). We found a significant association of DKD with rs3116494 in the *CD28* gene region (*P* = 0.0360) and a marginal association with rs3850890 in the *CD80* gene region (*P* = 0.0929) in stage 1. In stage 2, these SNPs, rs3116494 (*P* = 0.0116) and rs3850890 (*P* = 0.0382), were also associated with DKD. We then performed meta-analysis for rs3116494 and rs3850890 to combine the results of the two stages; as shown in Figures 1(d) and 1(e), under a fixed effects model (*P*_for heterogeneity_ = 0.6961), rs3116494 was significantly associated with DKD (OR = 1.29 [95% CI 1.11, 1.51], *P* = 0.0011). Similarly, under a fixed effects model (*P*_for heterogeneity_ = 0.1279), rs3850890 was significantly associated with DKD (OR = 1.16 [95% CI 1.02, 1.31], *P* = 0.0283). We further performed interaction analysis of *CD28*-rs3116494 and *CD80*-rs3850890 with DKD and found a risk-allele dose-dependent effect on DKD occurrence (*P* = 0.0098); subjects carrying the rs3116494 G allele and rs3850890 TT genotype showed the highest prevalence of DKD (Figure 1(f)).

### 3.3. Association of Validated SNPs with DKD-Related Traits

We tested the association of the validated SNPs with DKD-related traits, including serum creatinine, eGFR, and uAER. As shown in Table 2, the presence of the minor allele G of rs3116494 and the minor allele C of rs3850890 exhibited a trend towards increased serum creatinine and uAER and decreased eGFR; the direction of these effects was similar to those of DKD risk, although we did not have significant results. Although we obtained similar results in stage 2, the statistical power was limited. Therefore, we conducted a meta-analysis to combine the results; there was no heterogeneity detected in the two stages (*P*_for heterogeneity_ > 0.05). The meta-analysis results showed that both rs3116494 (*β* = −3.91, SE = 2.41, *P* = 0.0181) and rs3850890 (*β* = −2.72, SE = 1.02, *P* = 0.0077) were significantly associated with eGFR, and rs3850890 was associated with uAER (*β* = 2.80, SE = 1.18, *P* = 0.0178).

### 3.4. Serum sCD28 Levels in DKD and Control Groups

**Figure 2:**
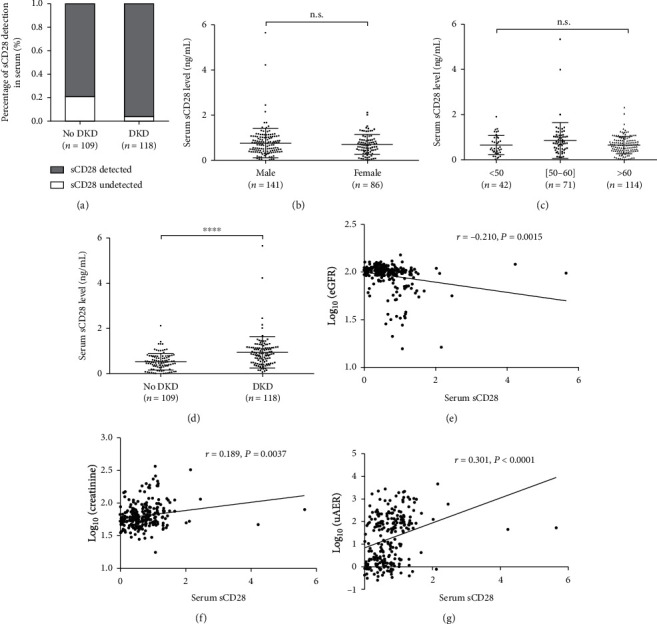
Serum sCD28 levels in patients with T2DM and its association with DKD and related traits. (a) Owing to limited assay sensitivity, sCD28 was not detected in several patients (21% in the control group and 4% in the DKD group). (b) Comparison of serum sCD28 levels in male and female patients in the study population (*P* > 0.05). (c) Comparison of serum sCD28 levels in different age groups (*P* > 0.05). (d) Comparison of serum sCD28 levels in DKD and control groups. (e) Relationship between serum sCD28 levels and log-transformed eGFR (*r* = −0.210, *P* = 0.0015). (f) Relationship between serum sCD28 levels and log-transformed creatinine (*r* = 0.189, *P* = 0.0037). (g) Relationship between serum sCD28 levels and log-transformed urinary albuminuria excretion rate (*r* = 0.301, *P* < 0.0001). n.s. means not significant (*P* > 0.05); ^∗∗∗∗^*P* < 0.0001.

**Figure 3:**
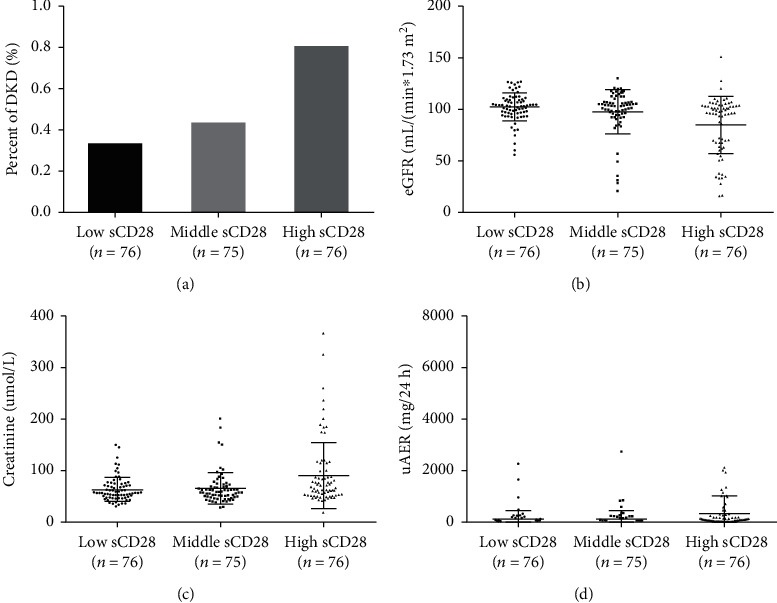
DKD and related traits in patients with T2DM based on different serum sCD28 levels. Comparison of (a) DKD occurrence (*P*_trend_ < 0.0001), (b) eGFR (*P*_trend_ < 0.0001), (c) serum creatinine (*P*_trend_ < 0.0001), and (d) urinary albuminuria excretion rate (*P*_trend_ < 0.0001) based on the serum sCD28 tertiles: low sCD28 (0.00-0.48 ng/mL), middle sCD28 (0.49-0.87 ng/mL), and high sCD28 (0.87-5.65 ng/mL). *P*_trend_ values were determined using logistic regression to test dose-response relationships for different traits according to serum sCD28 tertiles.

We referred to the transcriptomic database obtained from renal tissue from subjects with DKD and controls (http://www.nephroseq.org) and found that the CD28 expression was higher in the kidneys of patients with DKD (fold change = 1.881, *P* = 0.011), but *CD80* and *CTLA4* expression showed no differences. We further explored the role of plasma sCD28 protein in DKD. The demographic and clinical characteristics of these 227 patients are presented in Supplementary Table 2. Similar to the total study population, duration of T2DM and HbA1c levels were higher in control than those in the DKD group. Owing to the limited sensitivity of ELISA, sCD28 could not be detected in all individuals, 21% in T2DM without DKD and 4% in T2DM with DKD (Figure 2(a)). Serum sCD28 levels did not differ based on the sex (Figure 2(b)) as well as the age (Figure 2(c)) of patients. Further, we found that the control group, comprising patients with long-term T2DM without DKD and DR, showed significantly lower levels of serum sCD28 compared to that in the DKD group (*P* < 0.0001) (Supplementary Table 2). We then stratified the individuals into tertiles based on sCD28 levels (low sCD28 [0.00-0.48 ng/mL], middle sCD28 [0.49-0.87 ng/mL], and high sCD28 [0.87-5.65 ng/mL]) and found that patients having high plasma sCD28 levels had the highest DKD risk; a total of 0.8% in the high sCD28 group but only 0.33% in the low sCD28 group had DKD (Figure 3(a)).

### 3.5. Association of Serum sCD28 Levels with DKD-Related Traits

Pearson's correlation analysis revealed that serum sCD28 levels were significantly associated with DKD-related traits: eGFR (*r* = −0.210, *P* = 0.0015), plasma creatinine (*r* = −0.210, *P* = 0.0015), and uAER (*r* = −0.210, *P* = 0.0015) (Figures 2(e)–2(g)). However, according to Spearman's rank correlation analysis, sCD28 was not associated with age, gender, BMI, duration of diabetes, and HbA1c, while significant results similar to those obtained by Pearson's correlation analysis were observed for DKD-related traits (Supplementary Table 3). Further, we performed multiple linear regression analysis between sCD28 levels and DKD-related traits after adjusting for the confounding factors of age, gender, and BMI in model 1 and age, gender, BMI, duration of diabetes, and HbA1c in model 2; the results for both statistical models were significant (Supplementary Table 3): serum creatinine (*P* = 0.0032, *P* = 0.0037; respectively), eGFR (*P* = 0.0002, *P* = 0.0002; respectively), and uAER (*P* = 0.0039, *P* = 0.0039; respectively). We also analyzed DKD-related traits according to the sCD28 tertiles. Trend tests using logistic regressions were conducted to assess the associations. The results showed the highest levels of plasma creatinine (*P*_trend_ < 0.0001), highest uAER (*P*_trend_ < 0.0001), and lowest eGFR (*P*_trend_ < 0.0001) were in the high sCD28 group (Figures 3(b)–3(d)).

### 3.6. Association of Validated SNPs with Serum sCD28 Levels and Mendelian Randomization Analysis

**Figure 4:**
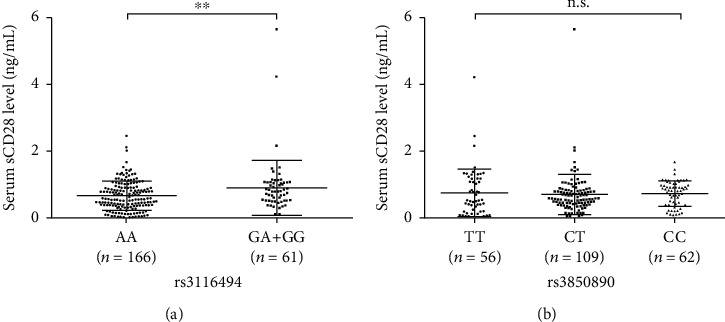
Association of *CD28*-rs3116494 and *CD80*-rs3850890 genotypes with serum sCD28 levels. (a) Comparison of serum sCD28 levels in *CD28*-rs3116494 genotype groups; as percentage of subjects with the GG genotype was low, we combined individuals with the GA and the GG genotypes as one group. *P* values were determined using *t*-tests (*P* = 0.0091). (b) Comparison of serum sCD28 levels in different *CD80*-rs3850890 genotype groups. *P* values were determined by one-way ANOVAs (*P* > 0.05). n.s. means not significant (*P* > 0.05); ^∗∗^*P* < 0.01.

The association of validated SNPs with serum sCD28 levels was also analyzed in this study. rs3116494 was associated with serum sCD28 level (*β* = 0.26 [95% CI 0.08, 0.44], *P* = 0.0043). As the percentage of subjects with the rs3116494 GG genotype was low, we combined GA and GG genotypes as one group. As shown in Supplementary Figure 1, individuals carrying the rs3116494 G allele had higher serum sCD28 levels compared to the carriers of A allele homozygosis (*P* < 0.01); however, the genotypes of rs3850890 were not associated with serum sCD28 levels (Figure 4). Mendelian randomization analysis using inverse-variance weighted methods indicated that elevated levels of the circulating costimulatory molecule sCD28 play a causal role in DKD (*β* = 1.15 [95% CI 0.46, 1.83], *P* = 0.0010).

## 4. Discussion

In the present study, we analyzed the causal relationship of the circulating costimulatory molecule sCD28 and its related genetic variants with DKD. We divided the patients into DKD and control groups. Notably, the controls had higher duration of the T2DM and did not have DR. Thus, control individuals were relatively resistant to DKD. Therefore, a genetic association study in our sample population may help to detect the genetic loci associated with DKD susceptibility. We identified 2 novel SNPs (rs3116494 and rs3850890) that were significantly associated with DKD susceptibility. Furthermore, the association of the two SNPs with the occurrence of DKD in the present study suggested the potential interactive role of CD28 and CD80 in DKD. Additionally, Nephroseq analysis revealed that CD28, but not CD80 and CTLA4, was differentially expressed in the kidneys of patients with DKD compared to that in the healthy controls. Therefore, we further analyzed sCD28 protein levels in circulation with respect to DKD susceptibility and related traits. We found a significantly lower level of sCD28 in controls than in the DKD group. Additionally, serum sCD28 level was significantly associated with serum creatinine, eGFR, and uAER. Moreover, the *CD28*-rs3116494 genotype was also found to be significantly associated with sCD28 levels. Mendelian randomization analysis indicated that rs3116494 played a causal role in DKD by influencing serum sCD28 levels.

We conducted the present study in two stages to determine the genetic association; we found that the patients with rs3116494-G or rs3850890-C alleles had a higher risk for DKD. Through interactive analysis, we also found the two SNPs were interactive on DKD. Patients with the AA genotype of rs3116494 and the CC genotype of rs3850890 had the lowest prevalence of DKD, while carriers of the rs3116494 G allele and the rs3850890 T allele homozygosis had the highest prevalence. This suggested that CD28 and CD80 have a combined role in DKD. Further, we also analyzed the association of these SNPs with eGFR, uAER, and creatinine, which are widely considered biomarkers for the DKD phenotype. We found that rs3116494-G was associated with decreased eGFR and increased serum creatinine, while rs3850890-C was associated with decreased eGFR and increased uAER. These findings verified that the DKD risk alleles of the two SNPs were associated with impaired kidney function in patients with T2DM.

More DKD- and kidney function-related genetic variants have been discovered in recent years. However, most loci were located in noncoding regions, and the concerned biological explanations remain elusive. Gene expression and tissue-specific eQTL data are powerful tools to obtain functional annotations for these variants. Thus, we analyzed gene expression and eQTL data according to Nephroseq and GTEx databases. According to the Nephroseq, we found that expression of *CD28* was higher in kidney biopsy samples of patients with DKD than in those of healthy living donors (fold change = .881, *P* = 0.011), while the expression of *CD80* and *CTLA4* showed no difference (*P* > 0.05). Increased CD28 expression in kidneys might be associated with T cell infiltration in patients with DKD. Furthermore, to better understand the SNPs in the present genetic association study, we performed eQTL analysis according to the GTEx Portal [34, 35]. Based on the single-tissue eQTL map (Figure S1), rs3116494-G (DKD risk allele in our study) was associated with an increased CD28 expression in most of the tissues including the tibial artery (NES = 0.147, *P* = 0.0002), coronary artery (NES = 0.142, *P* = 0.008), heart (NES = 0.123, *P* = 0.02), visceral adipose (NES = 0.109, *P* = 0.005), and small intestine in the terminal ileum (NES = 0.0748, *P* = 0.04). Additionally, rs3116494 was also identified by the genome-wide association studies in inflammatory bowel disease (OR = 1.06, *P* = 5 × 10^−7^, *n* = 27432) and ulcerative colitis (OR = 1.08, *P* = 1 × 10^−7^, *n* = 34652), where the G allele carriers had higher risk for both diseases [36, 37]. These results suggested an autoimmunity regulatory function for rs3116494. The SNP was also marginally associated with CD28 expression in the whole blood (NES = 0.0304, *P* = 0.06). However, we did not find an association between rs3850890 and *CD80* expression using the GTEx Portal (expression data in bone marrow or lymphoid tissue was unavailable). Besides, we did not find an association between rs3116494 and *CD28* gene expression in kidneys. Our results suggested that rs3116494 might not influence the *CD28* gene expression in the kidneys but may do so in other tissues like the artery, visceral adipose, and blood. Thus, the association of rs3116494 with DKD may be attributed to system immunology.

We further explored the role of serum sCD28 protein level in DKD in a subset of patients with T2DM. The patient characteristics in the DKD and control groups were similar to those in the total study population. Significantly lower sCD28 levels were observed in patients with long-term T2DM without DKD and DR as compared to those with DKD. The results suggested that low level of sCD28 in circulation might be responsible for DKD tolerance. Additionally, sCD28 levels were positively associated with serum creatinine and uAER and negatively associated eGFR. We also observed an association of rs3116494 with serum sCD28 levels in our population; the G allele carriers had higher sCD28 levels. As independent analyses showed that the rs3116494 G allele and increased sCD28 levels were associated with a high risk of DKD and that the rs3116494 G carriers had higher sCD28 levels, we further performed a Mendelian randomization analysis that is a widely used approach to determine causal relationship in a cross-sectional study. Considering rs3116494 as a genetic tool and serum sCD28 as an intermediate variable, we analyzed the causation relationship between serum sCD28 and DKD. The results showed a significant causal relationship of sCD28 in DKD. This further verified the role of CD28 in human DKD.

The strengths of this study were that obviously disease susceptibility differs depending on whether the cases were DKD or control. The study had certain limitations: first, we did not have available data on medications including ACEI/ARB inhibitors, and these factors may influence the phenotype of DKD. Second, we could not assess the T cell immunity status of in the study population as the relevant data was not available. Third, lifestyle factors were not considered in this study.

## 5. Conclusions

In conclusion, the present study identified novel DKD-related SNPs in the *CD28* gene region in a Chinese population. Moreover, based on the method of Mendelian randomization analysis, our study provided evidence for a causal relationship between circulating costimulatory molecular sCD28 and DKD.

## Figures and Tables

**Table 1:** Demographic and clinical characteristics of study participants.

Traits	Stage 1 (*n* = 575)			Stage 2 (*n* = 1382)			Unselected T2DM patients for DKD-related traits (*n* = 1296)
	DKD	Control	*P* value	DKD	Control	*P* value	
Male/female	126/78	193/178	0.0245	591/228	315/248	<0.0001	632/664
Age (years)	56.29 ± 10.82	63.60 ± 10.90	<0.0001	48.95 ± 9.72	64.47 ± 10.75	<0.0001	60.70 ± 12.19
Body mass index (kg/m^2^)	25.84 ± 4.07	24.32 ± 3.59	<0.0001	25.42 ± 3.57	24.24 ± 3.29	<0.0001	25.23 ± 6.19
Duration of diabetes (years)	10.00 (5.00, 15.00)	12.00 (10.00, 17.00)	<0.0001	10.00 (4.00, 14.00)	14.00 (10.00, 17.00)	<0.0001	7.00 (3.00, 12.00)
HbA1c (%)	8.90 (7.00, 9.90)	9.10 (7.50, 10.20)	0.0505	7.10 (6.30, 8.40)	8.20 (7.10, 9.50)	<0.0001	8.00 (6.90, 9.80)
DR (%)	36.70%	0%	—	34.62%	0%	—	25.92%
Plasma creatinine (*μ*mol/L)	76.00 (59.00, 103.00)	56.00 (50.00, 66.00)	<0.0001	121.00 (84.80, 168.00)	63.50 (55.00, 73.00)	<0.0001	65.00 (54.00, 78.00)
eGFR (mL/(min·1.73 m^2^))	85.01 (72.30, 95.01)	103.19 (93.88, 112.11)	<0.0001	83.06 (72.13, 95.00)	106.32 (97.49, 114.90)	<0.0001	98.40 (86.82, 107.97)
Urinary albumin excretion rate (mg/24 h)	128.13 (68.69, 315.00)	7.68 (2.88, 14.54)	<0.0001	106.46 (72.14, 295.21)	9.37 (3.53, 16.12)	<0.0001	18.12 (6.45, 30.23)

Data are shown as mean ± standard deviation or median (interquartile range). The chi-square test was used to analyze the proportion. Student's *t*-test was used for normally distributed traits. The Wilcoxon test was used for skewed distributed traits. *P* < 0.05 was considered statistically significant. HbA1c: hemoglobin A1c; DR: diabetic retinopathy; eGFR: estimated glomerular filtration rate.

**Table 2:** Association between validated SNPs and DKD-related traits in study participants.

SNP	Chr	Gene	Position (building 37)	Minor/major allele	Traits	Stage 1 (*n* = 575)			Stage 2 (*n* = 2678)			Meta-analysis		
						*β*	SE	*P* value	*β*	SE	*P* value	*β*	SE	*P* value
rs3116494	2	CD28	204592021	G/A	Creatinine	3.34	2.47	0.1763	5.59	3.54	0.1143	4.08	2.03	0.0442
					eGFR	-3.27	1.89	0.0836	-3.91	2.41	0.1047	-3.51	1.49	0.0181
					uAER	10.86	7.34	0.1390	2.75	2.08	0.1861	3.35	2.00	0.0939

rs3850890	3	CD80	119265619	C/T	Creatinine	2.16	1.68	0.1985	1.88	1.96	0.3375	2.04	1.28	0.1095
					eGFR	-2.37	1.57	0.1312	-2.97	1.34	0.0267	-2.72	1.02	0.0077
					uAER	19.94	11.16	0.0740	2.61	1.19	0.0283	2.80	1.18	0.0178

*P* values were calculated by multiple linear regression under an additive genetic model adjusted for age, gender, body mass index, duration of diabetes, and HbA1c; the effect values were evaluated by minor allele. *P* < 0.05 was considered statistically significant. Chr: chromosome; eGFR: estimated glomerular filtration rate; uAER: urinary albumin excretion rate.

## Data Availability

All data relevant to the study are included in the article or uploaded as supplementary information.

## Supplementary Materials

Supplementary Materials Supplementary Figure 1: single-tissue eQTL map of rs3116494 with expression of CD28 in the GTEx database. Supplementary Table 1: association of SNPs in CD28, CTLA4, and B7-1 (CD80) with DKD. Supplementary Table 2: demographic and clinical characteristics of 227 patients included for serum sCD28 level determination. Supplementary Table 3: association of serum sCD28 level with clinical traits.
