# Supplementary material for: CD28 Genetic Variants Increase Susceptibility to Diabetic Kidney Disease in Chinese Patients with Type 2 Diabetes: A Cross-Sectional Case Control Study
Source: Mediators Inflamm. 2021 Apr 17;2021:5521050. doi: 10.1155/2021/5521050 (PMC8075672; doi:10.1155/2021/5521050)
Supplement: Supplementary Materials — Supplementary Figure 1: single-tissue eQTL map of rs3116494 with expression of CD28 in the GTEx database. Supplementary Table 1: association of SNPs in CD28, CTLA4, and B7-1 (CD80) with DKD. Supplementary Table 2: demographic and clinical characteristics of 227 patients included for serum sCD28 level determination. Supplementary Table 3: association of serum sCD28 level with clinical traits. [file 5521050.f1.zip › 5521050.f1.pdf]

Figure S1. Single-tissue eQTLs map of rs3116494 with expression of CD28 in GTEx database

Tissue	Samples	NES	p-value	m-value
Brain - Substantia nigra	114	0.263	0.07	0.852
Minor Salivary Gland	144	0.153	0.08	0.866
Artery - Tibial	584	0.147	2.0e-4	0.996
Artery - Coronary	213	0.142	0.008	0.945
Brain - Spinal cord (cervical c-1)	126	0.139	0.2	0.694
Heart - Atrial Appendage	372	0.123	0.02	0.925
Esophagus - Muscularis	465	0.121	0.03	0.947
Heart - Left Ventricle	386	0.121	0.04	0.907
Testis	322	0.118	0.1	0.862
Brain - Hypothalamus	170	0.117	0.3	0.694
Adipose - Visceral (Omentum)	469	0.109	0.005	0.978
Pituitary	237	0.107	0.1	0.787
Muscle - Skeletal	706	0.0884	0.06	0.892
Breast - Mammary Tissue	396	0.0881	0.04	0.885
Adrenal Gland	233	0.0817	0.4	0.675
Artery - Aorta	387	0.0790	0.08	0.892
Small Intestine - Terminal Ileum	174	0.0748	0.04	0.905
Ovary	167	0.0716	0.4	0.661
Nerve - Tibial	532	0.0691	0.1	0.883
Esophagus - Gastroesophageal Junction	330	0.0669	0.3	0.746
Stomach	324	0.0584	0.3	0.694
Cells - EBV-transformed lymphocytes	147	0.0412	0.7	0.599
Lung	515	0.0396	0.2	0.542
Colon - Sigmoid	318	0.0382	0.5	0.665
Whole Blood	670	0.0304	0.06	0.248
Adipose - Subcutaneous	581	0.0268	0.4	0.402
Vagina	141	0.0206	0.8	0.589
Colon - Transverse	368	0.0140	0.7	0.317
Brain - Amygdala	129	-	-	-
Brain - Anterior cingulate cortex (BA24)	147	-	-	-
Brain - Caudate (basal ganglia)	194	-	-	-
Brain - Cerebellar Hemisphere	175	-	-	-
Brain - Cerebellum	209	-	-	-
Brain - Cortex	205	-	-	-
Brain - Frontal Cortex (BA9)	175	-	-	-
Brain - Hippocampus	165	-	-	-
Brain - Nucleus accumbens (basal ganglia)	202	-	-	-
Brain - Putamen (basal ganglia)	170	-	-	-
Cells - Cultured fibroblasts	483	-	-	-
Pancreas	305	-	-	-
Prostate	221	-0.000283	1	0.452
Spleen	227	-0.00212	1	0.471
Kidney - Cortex	73	-0.0119	0.9	0.555
Skin - Sun Exposed (Lower leg)	605	-0.0139	0.7	0.0930
Thyroid	574	-0.0227	0.5	0.0510
Skin - Not Sun Exposed (Suprapubic)	517	-0.0231	0.6	0.161
Uterus	129	-0.0386	0.7	0.459
Esophagus - Mucosa	497	-0.0720	0.01	0.00300
Liver	208	-0.0776	0.3	0.262

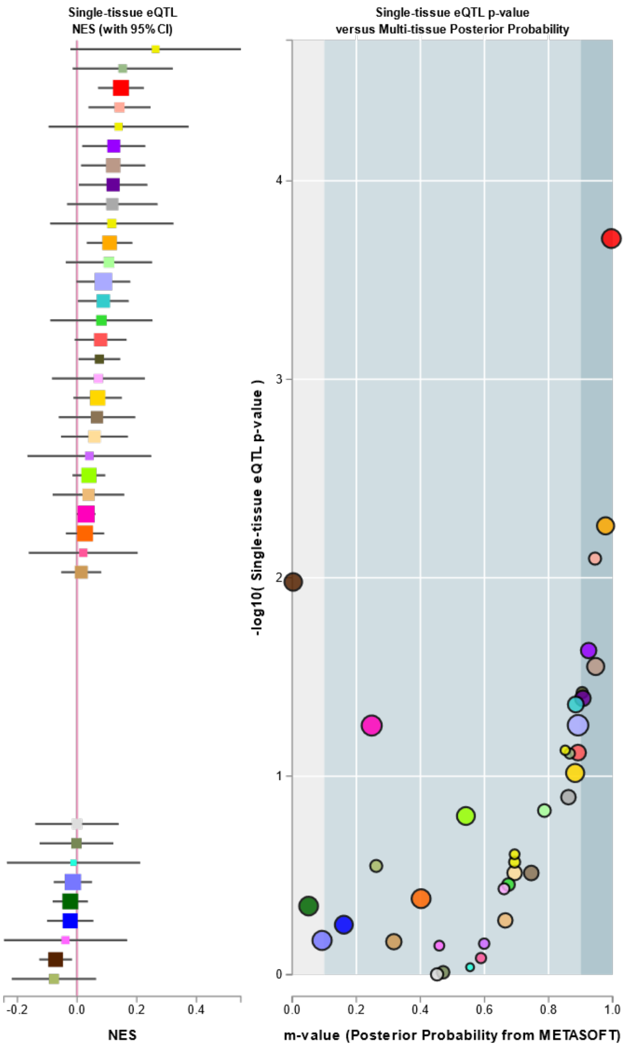

NES: the slope of linear regression of normalized expression data with rs3116494 genotypes using single-tissue eQTL analysis, representing an eQTL effect size. m-value refers to the probability that an eQTL effect exists in each tissue tested in the cross-tissue meta-analysis.
