# Supplementary material for: CD28 Genetic Variants Increase Susceptibility to Diabetic Kidney Disease in Chinese Patients with Type 2 Diabetes: A Cross-Sectional Case Control Study
Source: Mediators Inflamm. 2021 Apr 17;2021:5521050. doi: 10.1155/2021/5521050 (PMC8075672; doi:10.1155/2021/5521050)
Supplement: Supplementary Materials — Supplementary Figure 1: single-tissue eQTL map of rs3116494 with expression of CD28 in the GTEx database. Supplementary Table 1: association of SNPs in CD28, CTLA4, and B7-1 (CD80) with DKD. Supplementary Table 2: demographic and clinical characteristics of 227 patients included for serum sCD28 level determination. Supplementary Table 3: association of serum sCD28 level with clinical traits. [file 5521050.f1.zip › 5521050.f2.docx]

Supplementary Table 1

Association of SNPs in CD28, CTLA4, and B7-1(CD80) with DKD

| Gene | SNP | Chr | Position (Building 37) | Minor/ major allele | DKD  MAF | Control MAF | OR (95% CI) | *P*_additive_  value |
| --- | --- | --- | --- | --- | --- | --- | --- | --- |
| CD28 | rs3181100 | 2 | 204572006 | G/C | 0.164 | 0.158 | 0.96(0.78,1.21) | 0.1041 |
|  | rs151302357 | 2 | 204574597 | A/G | 0.027 | 0.026 | 1.18(0.69,1.99) | 0.2615 |
|  | rs150173965 | 2 | 204580773 | G/A | 0.018 | 0.017 | 1.43(0.74,2.75) | 0.2881 |
|  | rs3769684 | 2 | 204584759 | T/C | 0.472 | 0.443 | 1.15(0.97,1.36) | 0.9686 |
|  | rs3116494 | 2 | 204592021 | G/A | 0.105 | 0.090 | 1.35(1.02,1.80) | **0.0360** |
|  | rs143570892 | 2 | 204596488 | A/C | 0.011 | 0.013 | 1.04(0.83,1.31) | 0.8375 |
|  | rs45620941 | 2 | 204600457 | T/G | 0.101 | 0.097 | 1.042(0.83,1.31) | 0.7301 |
| CTLA4 | rs231775 | 2 | 204732714 | A/G | 0.319 | 0.327 | 0.98(0.84,1.13) | 0.7504 |
|  | rs117492436 | 2 | 204733098 | T/A | 0.006 | 0.008 | 0.60(0.26,1.37) | 0.2252 |
|  | rs231777 | 2 | 204733588 | T/C | 0.133 | 0.131 | 0.94(0.77,1.15) | 0.5538 |
|  | rs35219727 | 2 | 204734049 | A/G | 0.018 | 0.023 | 0.70(0.43,1.14) | 0.1533 |
|  | rs55657178 | 2 | 204735201 | C/G | 0.007 | 0.008 | 0.86(0.38,1.96) | 0.7186 |
|  | rs56102377 | 2 | 204737635 | A/G | 0.036 | 0.045 | 0.82(0.58,1.16) | 0.2624 |
|  | rs55696217 | 2 | 204738577 | A/G | 0.012 | 0.012 | 1.03(0.53,1.97) | 0.9390 |
| B7-1(CD80) | rs17281703 | 3 | 119243549 | A/G | 0.102 | 0.093 | 1.05(0.83,1.31) | 0.6968 |
|  | rs9877854 | 3 | 119245747 | G/T | 0.010 | 0.097 | 0.91(0.72,1.14) | 0.4002 |
|  | rs2629396 | 3 | 119248967 | G/T | 0.399 | 0.407 | 0.98(0.85,1.12) | 0.7591 |
|  | rs1797839 | 3 | 119249871 | T/C | 0.098 | 0.096 | 0.93(0.74,1.17) | 0.5097 |
|  | rs75906281 | 3 | 119254782 | G/A | 0.168 | 0.192 | 0.86(0.72,1.03) | 0.1031 |
|  | rs28653390 | 3 | 119259163 | A/G | 0.168 | 0.192 | 0.87(0.73,1.04) | 0.1298 |
|  | rs693640 | 3 | 119264382 | T/C | 0.319 | 0.311 | 1.08(0.93,1.24) | 0.3123 |
|  | rs3850890 | 3 | 119265619 | C/T | 0.493 | 0.470 | 1.12(0.98,1.28) | **0.0929** |
|  | rs527004 | 3 | 119269112 | A/G | 0.119 | 0.106 | 1.11(0.90,1.38) | 0.3260 |
|  | rs2222631 | 3 | 119272391 | G/A | 0.473 | 0.453 | 1.06(0.93,1.21) | 0.3901 |
|  | rs184625151 | 3 | 119276418 | C/T | 0.010 | 0.012 | 0.73(0.38,1.42) | 0.3575 |
|  | rs16829980 | 3 | 119278468 | G/A | 0.241 | 0.250 | 0.90(0.77,1.05) | 0.1911 |

*P* values were calculated by logistic regression under additive genetic model adjusted for age, gender, body mass index, duration of diabetes, and HbA1c; the effect values were evaluated by minor allele. *P* < 0.1 was considered statistically significant.

Abbreviations: Chr, chromosome; MAF, minor allele frequency.

Supplementary Table 2

Demographic and clinical characteristics of 227 patients included for serum sCD28 level determination

| Traits | DKD | Control | *P* value |
| --- | --- | --- | --- |
| Male/female | 77/41 | 64/45 | 0.3103 |
| Age (years) | 58.45±10.63 | 59.71±9.64 | 0.3469 |
| Body mass index (kg/m^2^) | 25.98±3.91 | 24.64±3.99 | **0.0338** |
| Duration of diabetes (years) | 10.00 (6.00,15.00) | 13.00 (10.00,18.00） | **<0.0001** |
| HbA1c (%) | 9.10 (7.70, 10.10) | 9.40 (8.30, 10.60) | 0.0705 |
| DR (%) | 36.60% | 0% | - |
| sCD28 (ng/mL) | 0.87 (0.51,1.15) | 0.51 (0.29,0.77) | **<0.0001** |
| Plasma creatinine (μmol/L) | 74.50 (58.00,103.00) | 52.00 (45.00,61.00) | **<0.0001** |
| eGFR (mL/(min·1.73 m^2^)) | 94.40 (67.59, 103.63) | 104.59 (99.56, 109.21) | **<0.0001** |
| Urinary albumin excretion rate (mg/24 h) | 123.97 (69.4, 278.10) | 1.47 (0.81,3.18) | **<0.0001** |

Data are shown as mean ± standard deviation or median (interquartile range). The chi-square test was used to analysis the proportion, the t-test was used for normal distributed traits, and the Wilcoxon test was used for skewed distributed traits. P < 0.05 was considered statistically significant.

Abbreviations: HbA1c, hemoglobin A1c; DR, diabetic retinopathy; eGFR, estimated glomerular filtration rate.

Supplementary Table 3

Association of serum sCD28 level with clinical traits

|  | Spearman’s rank correlation | | Multiple linear regression model 1 | | Multiple linear regression model 2 | |
| --- | --- | --- | --- | --- | --- | --- |
| Parameters | r | *P* value | β (se) | *P* value | β (se) | *P* value |
| Age (years) | -0.026 | 0.8057 | - | - | - | - |
| Gender | -0.016 | 0.6936 | - | - | - | - |
| Body mass index (kg/m^2^) | 0.034 | 0.6007 | - | - | - | - |
| Duration of diabetes (years) | -0.103 | 0.1164 | -0.007 (0.007) | 0.2901 | - | - |
| HbA1c (%) | -0.057 | 0.3858 | 0.002 (0.018) | 0.9258 | - | - |
| Serum creatinine (μmol/L) | 0.225 | **0.0005** | 0.003 (0.001) | **0.0032** | 0.003 (0.001) | **0.0037** |
| eGFR (mL/ (min*1.73 m^2^) | -0.298 | **<0.0001** | -0.007 (0.002) | **0.0002** | -0.007 (0.002) | **0.0002** |
| Urinary albumin excretion rate (g/24 h) | 0.360 | **<0.0001** | 0.001 (0.000) | **0.0039** | 0.001 (0.000) | **0.0039** |

Multiple linear regression 1 was adjusted for age, gender, and body mass index; multiple linear regression 2 was adjusted for age, gender, body mass index, duration of diabetes, and HbA1c. Serum creatinine, eGFR, and urinary albumin excretion rate were log-transformed before analysis. *P* < 0.05 was considered statistically significant.

Abbreviations: HbA1c, hemoglobin A1c; eGFR, estimated glomerular filtration rate.
